# Differences in respiratory wheezing between current exclusive e-cigarette users, current exclusive cigarette smokers, and never users of either product: findings from a population-based study

**DOI:** 10.1186/s12954-025-01315-8

**Published:** 2025-10-03

**Authors:** Yusuff Adebayo Adebisi, Najim Z. Alshahrani, Lucia Spicuzza, Francesco Pennisi, Giulio Geraci, Giulio Giacono Cantone, Venera Tomaselli, Riccardo Polosa

**Affiliations:** 1 College of Social Sciences, University of Glasgow, 40 Bute Gardens, Glasgow, G12 8RT UK; 2 Department of Family and Community Medicine, Faculty of Medicine, University of Jeddah, Jeddah, Saudi Arabia; 3 Center of Excellence for the Acceleration of HArm Reduction (CoEHAR), University of Catania, Catania, Italy; 4 Department of Clinical & Experimental Medicine, University of Catania, Catania, Italy; 5 Respiratory Unit - University Teaching Hospital “Policlinico-S.Marco”, University of Catania, Catania, Italy; 6 Faculty of Medicine and Surgery, “Kore” University of Enna, Enna, Italy; 7 “Magna Graecia” University of Catanzaro, Catanzaro, Italy; 8 Centre for the Prevention and Treatment of Tobacco Addiction (CPCT), University Teaching Hospital “Policlinico-S.Marco”, University of Catania, Catania, Italy

**Keywords:** Wheezing, Respiratory symptoms, Exclusive cigarette smoking, Exclusive e-cigarette use

## Abstract

**Background:**

E-cigarettes have emerged as an alternative to combustible cigarettes, yet their comparative impact on respiratory symptoms remains uncertain. We investigated cross-sectional differences in self-reported wheezing between current exclusive e-cigarette users, current exclusive cigarette smokers, and never-users of either product.

**Methods:**

We analysed data from 9000 adults aged ≥ 16 years in the 2017–2019 Scottish Health Survey. The exposure was current nicotine use status (exclusive cigarette smoking, exclusive e-cigarette use, or never-use), and the outcome was self-reported wheezing in the past 12 months. Binary multivariable logistic regression estimated adjusted odds ratios (ORs) with 95% confidence intervals (CIs), controlling for age, sex, area-level socioeconomic deprivation, longstanding illness, doctor-diagnosed asthma and COPD, self-rated general health, alcohol consumption, age of smoking initiation, and exposure to second-hand smoke.

**Results:**

Compared to exclusive e-cigarette users, exclusive cigarette smokers had significantly higher odds of wheezing (adjusted OR = 1.80, 95% CI: 1.33–2.42, *p* < 0.001). Never-users had lower odds of wheezing than exclusive e-cigarette users, though the difference was not significant (adjusted OR = 0.66, 95% CI: 0.31–1.40, *p* = 0.275). Among exclusive cigarette smokers, the odds of wheezing increased with smoking intensity relative to exclusive e-cigarette users, indicating a dose-response relationship (χ² = 5.91, *p* = 0.018). Specifically, light smokers (< 10 cigarettes/day) had elevated but not significant odds (adjusted OR = 1.32, 95% CI: 0.92–1.89, *p* = 0.133), while moderate smokers (10–19 cigarettes/day) showed higher odds (adjusted OR = 1.85, 95% CI: 1.33–2.59, *p* < 0.001), and heavy smokers (≥ 20 cigarettes/day) had the greatest odds (adjusted OR = 2.27, 95% CI: 1.57–3.28, *p* < 0.001). Adjusted predicted probabilities of wheezing mirrored this pattern: compared with exclusive e-cigarette users, probabilities were significantly higher for moderate smokers (+ 7.2%, *p* = 0.002) and heavy smokers (+ 10.0%, *p* < 0.001), but not for light smokers (+ 3.0%, *p* = 0.152) or never-users (–3.9%, *p* = 0.306).

**Conclusions:**

Exclusive e-cigarette use was not associated with higher odds of wheezing compared with never-use, and it was linked to substantially lower odds than exclusive cigarette smoking. These findings suggest that, while complete abstinence remains the lowest-risk option, e-cigarette use may pose fewer respiratory symptoms than smoking, particularly for moderate-to-heavy smokers.

## Introduction

Cigarette smoking remains one of the leading preventable causes of disease and premature death worldwide. It is strongly linked to respiratory conditions such as asthma, and chronic obstructive pulmonary disease (COPD) [1–5]. Wheezing, a high-pitched whistling sound during breathing, is an important clinical marker of airway inflammation or obstruction and is often associated with reduced lung function and breathing difficulties [6, 7]. Numerous population-based studies show a clear dose–response relationship, with heavier cigarette consumption increasing the likelihood of persistent respiratory symptoms, including wheezing [8, 9]. Although smoking prevalence has declined in some regions following strong tobacco control policies, tobacco-related respiratory disease continues to pose a major public health burden, reinforcing the importance of evaluating both the risks of smoking and the potential of less harmful alternatives [10, 11].

In recent times, electronic nicotine delivery systems (ENDS), commonly known as e-cigarettes, have emerged as a widely discussed alternative to conventional tobacco products, frequently marketed as a tool for reducing harm or aiding in smoking cessation [12–14]. Unlike traditional cigarettes that combust tobacco to release smoke, e-cigarettes function by vaporising a nicotine-infused liquid, potentially lowering exposure to the numerous harmful chemicals found in tobacco smoke [15]. Despite this, the long-term impact of e-cigarette use on respiratory health remains uncertain. Preliminary research suggests that while vaping can cause airway irritation, oxidative damage, and lung inflammation, these effects are generally less pronounced than those linked to cigarette smoking [16–19]. Longitudinal findings from the US PATH Study further indicate that complete switching from cigarettes to e-cigarettes is associated with improvements in functionally important respiratory symptoms, comparable to those observed among people who quit tobacco altogether [16]. A recent narrative review of 12 prospective studies among individuals who had never smoked found that evidence for respiratory harm linked to exclusive e-cigarette use was limited [18]. Most studies (7 out of 12) reported no significant effects, and among the five that did, most findings were not consistent across analytical models [18]. However, the evidence base is dominated by US cohorts, with little population-based research from Europe.

To address this gap, we examined whether the likelihood of respiratory wheezing varies by nicotine use status, comparing exclusive e-cigarette users, exclusive cigarette smokers, and never-users of either product. Drawing on data from a large, population-based survey in Scotland, this study offers the first nationally representative European evidence on these associations, thereby contributing to debates on the respiratory effects of nicotine product use.

## Methods

### Data source, study design, and participants

This research uses secondary data from the 2017, 2018, and 2019 iterations of the Scottish Health Survey (SHeS), a nationally representative cross-sectional survey conducted annually to assess health behaviours and outcomes among individuals aged 16 years and older, as well as children under 16, residing in private households across Scotland [20]. The combined dataset includes responses from 18,971 participants—5300 from 2017, 6790 from 2018, and 6881 from 2019. Sampling was carried out using the postcode address file to ensure population representativeness. Data collection involved in-home interviews facilitated by computer-assisted personal interviewing (CAPI), with more sensitive topics addressed through self-completion questionnaires [21]. Each year featured an independently selected sample, with adult participants forming the analytic sample for this study. Reported response rates hovered around 50% in 2017 and 2018, with a slight dip to 49% in 2019 [20, 21]. The final merged dataset comprises individual-level demographic, lifestyle, behavioural, and health data derived from interviewer-administered components and self-reported instruments.

### Assessment of the main exposures: exclusive e-cigarette use, cigarette smoking, and never-use

After excluding children aged 0–15 years, the dataset comprised 13,410 adults. Cigarette smoking status was determined using variable rcigst1, which classified respondents as current smokers (*n* = 2282), former smokers (*n* = 3469), or never smokers (*n* = 7576). E-cigarette use was assessed using variable Ecigtot16, which categorised individuals as current users (*n* = 924), former users (*n* = 1370), or never users (*n* = 11,036).

To construct mutually exclusive exposure groups, we generated a new variable based on current use. Three categories were defined: current exclusive cigarette smokers (those who currently smoke cigarettes but do not use e-cigarettes, regardless of past e-cigarette use; *n* = 1923), current exclusive e-cigarette users (those who currently use e-cigarettes but do not smoke cigarettes, regardless of past smoking history; *n* = 565), and never-users (those who have never smoked cigarettes and never used e-cigarettes; *n* = 7365).

Participants who reported dual use (cigarettes and e-cigarettes), former use of either product without current use, or incomplete data (*n* = 3557) were excluded. The analytic sample therefore comprised 9853 adults distributed across the three exposure groups.

### Assessment of the main outcome: respiratory wheezing

The primary outcome was respiratory wheezing within the past 12 months. Among 9853 adults categorised into three mutually exclusive exposure groups, exclusive cigarette smokers, exclusive e-cigarette users, and never-users, we assessed wheezing using two questions. First, participants were asked whether they had ever experienced wheezing or whistling in the chest. Those who responded “yes” were then asked if this occurred in the past 12 months. Due to survey skip logic, participants who reported never wheezing were not asked the follow-up question and were logically classified as not having wheezed in the past year. Using these responses, a binary variable was created to capture past-year wheezing. Participants with missing or invalid responses, including routing errors, refusals, or non-responses, were excluded (*n* = 853), resulting in a final analytical sample of 9,000 adults with valid data for both exposure and outcome.

### Information on other covariates

We grouped age into seven categories to reflect varying respiratory risks across the lifespan: 16–24, 25–34, 35–44, 45–54, 55–64, 65–74, and 75 + years. Sex was classified as male or female. Socioeconomic deprivation was measured using the Scottish Index of Multiple Deprivation (SIMD) quintiles, from the most to the least deprived. The presence of a longstanding illness was captured through a binary (yes/no) response to whether individuals had a chronic condition lasting, or expected to last, 12 months or more. Self-reported medical diagnoses were used to identify cases of doctor-diagnosed asthma and COPD. General health was self-rated and grouped into very good/good, fair, or bad/very bad. Alcohol use over the past year was categorised as almost daily to weekly, monthly or occasional, or non-drinker. Among those who reported ever smoking cigarettes, smoking initiation age was split into early (≤ 16 years) and late starters (> 16 years), with non-responses recorded. Never-smokers were coded as not applicable for this variable. Exposure to second-hand smoke was determined by asking participants whether they were regularly exposed in the home or elsewhere (yes/no). These variables were chosen due to their known links with both respiratory health outcomes and nicotine consumption patterns [22–24].

### Statistical analyses

#### Main analyses

Descriptive statistics were computed to compare characteristics across the three main exposure groups: exclusive cigarette smokers, exclusive e-cigarette users, and never-users. Chi-square tests were used for categorical variables, with p-values reported to indicate whether observed differences in baseline characteristics across groups were statistically significant. This provides important context for the regression analyses by identifying potential imbalances that may confound associations. Fisher’s exact test was used when one or more expected cell counts in the contingency table were less than 5.

Binary logistic regression was used to examine the association between nicotine use status and respiratory wheezing in the past 12 months. To provide a clear baseline for comparison, we first estimated univariable logistic regression models, reporting crude odds ratios (ORs) with 95% confidence intervals (CIs). These unadjusted models show the raw associations between nicotine use and wheezing without accounting for confounding factors. We then fitted multivariable logistic regression models, adjusting for age group, sex, deprivation quintile, presence of longstanding illness, doctor-diagnosed COPD, doctor-diagnosed asthma, self-rated general health, alcohol consumption status, age of smoking initiation, and exposure to second-hand smoke. Reporting both crude and adjusted ORs allows us to distinguish between the unadjusted associations and those that persist after accounting for important sociodemographic and health-related confounders. Exclusive e-cigarette users were specified as the reference group in all regression models to enable direct comparison with both never-users and exclusive cigarette smokers.

To further evaluate dose-response relationships across nicotine use categories, a five-level exposure variable was constructed comprising: never-users, exclusive e-cigarette users (reference group), light smokers (< 10 cigarettes/day), moderate smokers (10–19 cigarettes/day), and heavy smokers (≥ 20 cigarettes/day). Both unadjusted and adjusted ORs were calculated for each category using logistic regression, and a test for trend was conducted to assess linear association across smoking intensity levels using likelihood ratio test. Adjusted predicted probabilities of wheezing were also derived from the multivariable model, with 95% CIs, and pairwise contrasts were performed to quantify differences between e-cigarette users and all other groups.

#### Supplementary analyses

First, we conducted two subgroup comparisons using multivariable logistic regression. Among exclusive e-cigarette users, we compared individuals who had never smoked with those who had previously smoked cigarettes but had switched to vaping (“switchers”). Separately, among exclusive cigarette smokers, we compared those who had previously vaped but returned to smoking (“switchers back to smoking”) with those who had never used e-cigarettes. Both models were adjusted for the same covariates as in the main analysis, except for age of smoking initiation, as this precedes and may influence switching behaviour rather than acting as a confounder of the current respiratory outcomes.

Second, we examined time since quitting smoking using multivariable logistic regression, reclassifying current nicotine use status into six categories: (i) exclusive cigarette smokers, (ii) exclusive e-cigarette users who quit smoking ≤ 5 years ago, (iii) exclusive e-cigarette users who quit 6–10 years ago, (iv) exclusive e-cigarette users who quit > 10 years ago, (v) exclusive e-cigarette users who had never smoked, and (vi) never-smokers who never vaped. This approach allowed us to assess whether wheezing declined with increasing time since quitting cigarettes, while also comparing exclusive e-cigarette users with and without prior smoking history against the clean reference group of never-smokers and never-vapers. Models were adjusted for the same covariates as in the main analysis, except for age of smoking initiation.

#### Sensitivity analyses

To further assess the robustness of our findings, we conducted a sensitivity analysis by re-estimating the main analyses after excluding individuals with self-reported doctor-diagnosed asthma or COPD, rather than statistically adjusting for these conditions. This aimed to verify whether the associations remained consistent when those with pre-existing respiratory diagnoses were removed. Additionally, to address possible year-to-year variation in the pooled 2017–2019 dataset, we included survey year as a covariate in all final regression models. Finally, a post-hoc power calculation was performed to determine whether the sample size in the main analysis was adequate to detect the observed differences in wheezing prevalence.

All analyses were conducted using Stata version 18, with a significance level set at *p* < 0.05.

## Results

A total of 9000 participants were included in the analysis. Of these, 6748 were never-users, 520 were exclusive e-cigarette users, and 1732 were exclusive cigarette smokers. The three groups differed significantly across a range of socio-demographic and health-related characteristics.

There were significant differences in age distribution (*p* < 0.001). Exclusive e-cigarette and cigarette users were more concentrated in the middle-aged categories (35–64 years), while never-users had the highest proportion of older adults (≥ 75 years, 12.4%). Sex distribution also differed (*p* < 0.001), with men comprising a larger proportion of exclusive nicotine product users (45.2% for e-cigarette users and 46.5% for cigarette smokers) compared to never-users (40.7%). Regarding socioeconomic status, differences by deprivation quintile were marked (*p* < 0.001). The prevalence of longstanding illness was highest among exclusive cigarette smokers (57.3%), followed by e-cigarette users (51.3%), and lowest among never-users (44.8%) (*p* < 0.001). A similar gradient was observed for doctor-diagnosed COPD (*p* < 0.001): 10.2% among cigarette smokers, 5.4% among e-cigarette users, and only 1.3% among never-users. Asthma prevalence was also significantly higher among cigarette smokers (19.2%) compared with never-users (15.2%), whereas it was lower among e-cigarette users (12.3%) (*p* < 0.001). Self-rated health differed significantly (*p* < 0.001), with never-users most likely to report good or very good health (76.7%), and cigarette smokers more likely to rate their health as bad or very bad (18.1%). Exposure to second-hand smoke was highest among cigarette smokers (53.1%), followed by e-cigarette users (33.3%) and never-users (21.0%) (*p* < 0.001). Finally, the prevalence of wheezing in the past 12 months was significantly higher among cigarette smokers (32.3%), followed by e-cigarette users (17.5%) and never-users (11.4%) (*p* < 0.001) (see Table 1).

**Table 1:** Participant characteristics by nicotine use status

Variable	Never users (*N* = 6748)	Exclusive E-cigarette users (*N* = 520)	Exclusive cigarette users (*N* = 1732)	All (*N* = 9000)	χ2, *P*-Value
Age group, n (%)					χ2 = 167.12, *p* < 0.001
16–24	581 (8.6)	26 (5.0)	135 (7.8)	742 (8.2)	
25–34	854 (12.7)	81 (15.6)	297 (17.2)	1232 (13.7)	
35–44	983 (14.6)	94 (18.1)	289 (16.7)	1366 (15.2)	
45–54	1149 (17.0)	123 (23.7)	359 (20.7)	1631 (18.1)	
55–64	1216 (18.0)	113 (21.7)	327 (18.9)	1656 (18.4)	
65–74	1129 (16.7)	73 (14.0)	220 (12.7)	1422 (15.8)	
75+	836 (12.4)	10 (1.9)	105 (6.1)	951 (10.6)	
Sex, n (%)					χ2 = 20.91, *p* < 0.001
Male	2748 (40.7)	235 (45.2)	805 (46.5)	3788 (42.1)	
Female	4000 (59.3)	285 (54.8)	927 (53.5)	5212 (57.9)	
Deprivation, n (%)					χ2 = 613.44, *p* < 0.001
Most Deprived	840 (12.5)	122 (23.4)	543 (31.4)	1505 (16.7)	
2	1198 (17.8)	147 (28.2)	437 (25.2)	1782 (19.8)	
3	1387 (20.5)	109 (21.0)	338 (19.5)	1834 (20.4)	
4	1629 (24.1)	71 (13.7)	239 (13.8)	1939 (21.5)	
Least Deprived	1694 (25.1)	71 (13.7)	175 (10.1)	1940 (21.6)	
Has longstanding illness, n (%)					χ2 = 88.49, *p* < 0.001
Yes	3026 (44.8)	267 (51.3)	992 (57.3)	4285 (47.6)	
No	3722 (55.2)	253 (48.7)	740 (42.7)	4715 (52.4)	
Doctor diagnosed COPD, n (%)					χ2 = 350.97, *p* < 0.001
Yes	90 (1.3)	28 (5.4)	177 (10.2)	295 (3.3)	
No	6658 (98.7)	492 (94.6)	1555 (89.8)	8705 (96.7)	
Doctor diagnosed Asthma, n (%)					χ2 = 21.23, *p* < 0.001
Yes	1027 (15.2)	64 (12.3)	332 (19.2)	1423 (15.8)	
No	5721 (84.8)	456 (87.7)	1400 (80.8)	7577 (84.2)	
Self-rated general health, n (%)					χ2 = 402.19, *p* < 0.001
Very good / good	5174 (76.7)	341 (65.6)	957 (55.2)	6472 (71.9)	
Fair	1181 (17.5)	119 (22.9)	462 (26.7)	1762 (19.6)	
Bad / very bad	393 (5.8)	60 (11.5)	313 (18.1)	766 (8.5)	
Alcohol consumption in the last 12 months, n (%)					χ2 = 7.74, *p* = 0.102
Almost daily to weekly drinking	3307 (49.0)	257 (49.4)	833 (48.2)	4397 (48.9)	
Monthly or occasional	2204 (32.7)	177 (34.1)	537 (31.1)	2918 (32.5)	
Non-drinker	1237 (18.3)	86 (16.5)	359 (20.7)	1676 (18.6)	
Age of smoking initiation, n (%)					Fisher exact *p* < 0.001
Early starters (≤ 16 years)	0 (0.00)	277 (53.2)	1032 (59.6)	1309 (14.5)	
Late starters (> 16 years)	0 (0.00)	189 (36.4)	686 (39.6)	875 (9.7)	
Missing/Not applicable	6748 (100.0)	54 (10.4)	14 (0.8)	6816 (75.7)	
Second-hand smoke, n (%)					χ2 = 716.66, *p* < 0.001
Not exposed	5333 (79.0)	347 (66.7)	812 (46.9)	6492 (72.1)	
Exposed	1415 (21.0)	173 (33.3)	920 (53.1)	2508 (27.9)	
Has wheezing in the last 12 months, n (%)					χ2 = 455.87, *p* < 0.001
Yes	769 (11.4)	91 (17.5)	560 (32.3)	1420 (15.8)	
No	5979 (88.6)	429 (82.5)	1172 (67.7)	7580 (84.2)	

In the unadjusted model, exclusive cigarette smokers had significantly higher odds of reporting wheezing in the past 12 months compared to exclusive e-cigarette users (OR = 2.25, 95% CI: 1.76–2.88, *p* < 0.001). Never users also had significantly lower odds of wheezing compared to e-cigarette users (OR = 0.61, 95% CI: 0.48–0.77, *p* < 0.001). After adjusting for age group, sex, area-level deprivation, longstanding illness, doctor-diagnosed COPD and asthma, self-rated general health, alcohol consumption, age of smoking initiation, and exposure to second-hand smoke (confounders), the association between exclusive cigarette use and wheezing remained statistically significant (adjusted OR = 1.80, 95% CI: 1.33–2.42, *p* < 0.001). However, the difference between never users and exclusive e-cigarette users was no longer statistically significant in the adjusted model (adjusted OR = 0.66, 95% CI: 0.31–1.40, *p* = 0.275) (see Table 2).Model fit statistics for the final model: McFadden’s pseudo-R² = 0.284; likelihood ratio χ² = 2228.86, p < 0.001.

**Table 2:** Crude and adjusted association between exclusive cigarette smokers, exclusive e-cigarette users, never users and respiratory wheezing

Model	Covariates included	Exclusive e-cigarette users	Never users	Exclusive cigarette smokers
Model 1	Crude	Reference	0.61 (0.48–0.77), *p* < 0.001	2.25 (1.76–2.88), *p* < 0.001
Model 2	+ Age group, Sex	Reference	0.60 (0.47–0.76), *p* < 0.001	2.29 (1.79–2.93), *p* < 0.001
Model 3	+ Deprivation	Reference	0.65 (0.51–0.83), *p* < 0.001	2.24 (1.75–2.88), *p* < 0.001
Model 4	+ Longstanding illness, COPD, Asthma	Reference	0.60 (0.46–0.80), *p* < 0.001	1.98 (1.48–2.63), *p* < 0.001
Model 5	+ General health, Alcohol, Age of smoking initiation	Reference	0.67 (0.32–1.42), *p* = 0.298	1.90 (1.41–2.56), *p* < 0.001
Final model	+ Second-hand smoke exposure	Reference	0.66 (0.31–1.40), *p* = 0.275	1.80 (1.33–2.42), *p* < 0.001

The analytic sample included 9000 participants, comprising 520 exclusive e-cigarette users, 6748 never users, and 1732 exclusive cigarette smokers. The outcome was self-reported respiratory wheezing in the past 12 months. Odds ratios (ORs) are presented with 95% confidence intervals. The final model additionally adjusted for second-hand smoke exposure. Model fit statistics for the final model: McFadden’s pseudo-R² = 0.284; likelihood ratio χ² = 2228.86, *p*< 0.001.

**Fig. 1:**
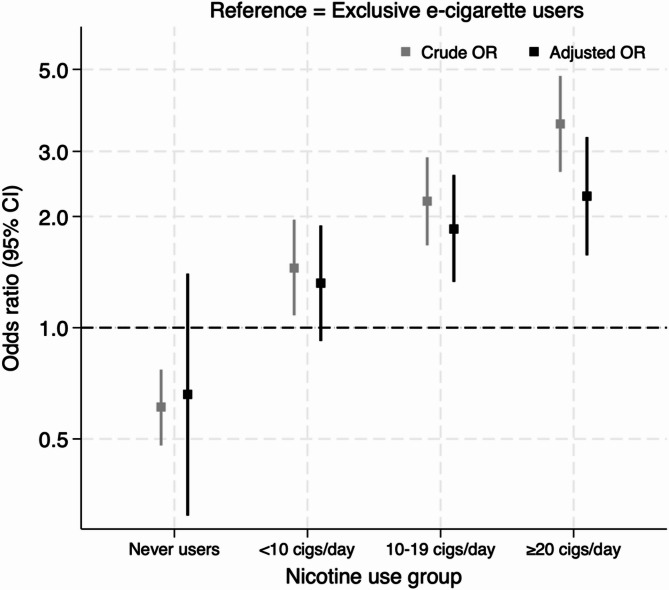
Crude and adjusted association between nicotine product use and respiratory wheezing. Sample sizes by nicotine use group were: Exclusive e-cigarette user (*n* = 520), Never smoker/e-cigarette users (*n* = 6,748), Light smoker < 10 cigarettes/day (*n* = 561), Moderate smoker 10–19 cigarettes/day (*n* = 708), and Heavy smoker ≥ 20 cigarettes/day (*n* = 407). A further 56 exclusive cigarette smokers with missing data on daily cigarette consumption were excluded from this model (analytic *N* = 8944). Adjusted estimates account for age group, sex, deprivation, longstanding illness, doctor-diagnosed COPD, doctor-diagnosed asthma, self-rated general health, alcohol consumption status, age of smoking initiation, and second-hand smoke exposure

Compared to exclusive e-cigarette users, the odds of wheezing increased with cigarette smoking intensity. In the unadjusted model, participants who smoked < 10 cigarettes/day (light smokers) (OR = 1.45, 95% CI: 1.08–1.96, *p* = 0.015), 10–19 cigarettes/day (moderate smokers) (OR = 2.20, 95% CI: 1.67–2.89, *p* < 0.001), and ≥ 20 cigarettes/day (heavy smokers) (OR = 3.56, 95% CI: 2.64–4.80, *p* < 0.001) had significantly higher odds of wheezing, whereas never users had lower odds (OR = 0.61, 95% CI: 0.48–0.77, *p* < 0.001). A strong linear trend was observed across smoking intensity categories (χ² = 343.39, *p* < 0.001). After adjustment for confounders, the associations remained significant for moderate smokers (10–19 cigs/day) (adjusted OR = 1.85, 95% CI: 1.33–2.59, *p* < 0.001) and heavy smokers (≥ 20 cigarettes/day) (adjusted OR = 2.27, 95% CI: 1.57–3.28, *p* < 0.001). By contrast, light smokers (< 10 cigarettes/day) no longer differed significantly from exclusive e-cigarette users (adjusted OR = 1.32, 95% CI: 0.92–1.89, *p* = 0.133), and never users also did not differ (adjusted OR = 0.66, 95% CI: 0.31–1.40, *p* = 0.275). A significant dose-response relationship remained (χ² = 5.91, *p* = 0.018), indicating that increasing cigarette consumption was associated with greater odds of wheezing (see Fig. 1).

**Fig. 2:**
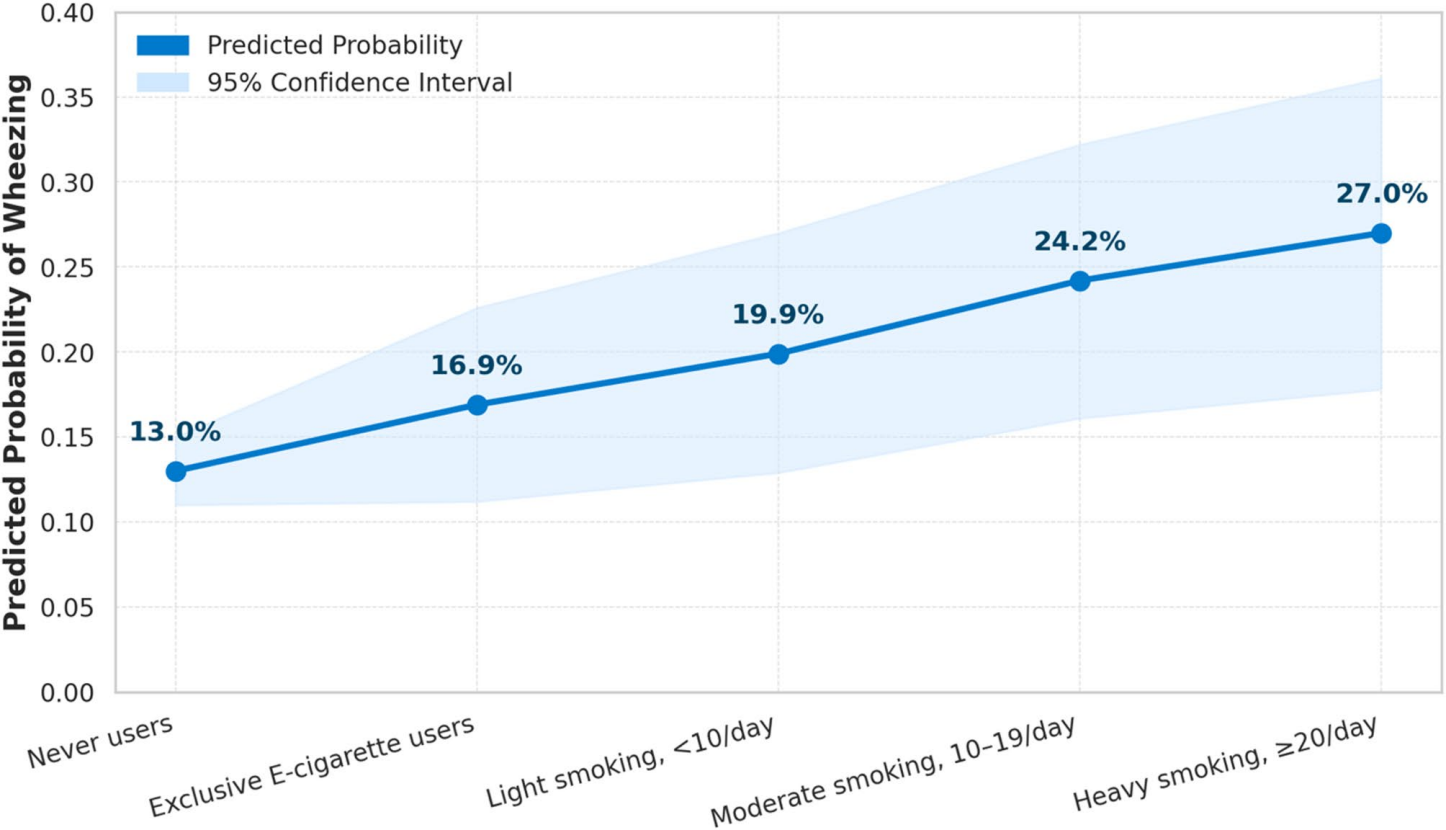
Adjusted predicted probability of respiratory wheezing by nicotine use group

Figure 2 displays the adjusted predicted probabilities of reporting wheezing in the past 12 months by nicotine use category. Never users had the lowest predicted probability (13.0%, 95% CI: 11.0–15.0%), followed by exclusive e-cigarette users (16.9%, 95% CI: 11.2–22.6%). The probability increased progressively with cigarette smoking intensity: 19.9% (95% CI: 12.9–27.0%) among light smokers, 24.2% (95% CI: 16.1–32.2%) among moderate smokers, and 27.0% (95% CI: 17.8–36.1%) among heavy smokers. Compared with exclusive e-cigarette users, the predicted probability of wheezing was significantly higher among moderate smokers (difference = + 7.2%, 95% CI: 2.7–11.8, *p* = 0.002) and heavy smokers (difference = + 10.0%, 95% CI: 4.5–15.6, *p* < 0.001), but not among light smokers (difference = + 3.0%, 95% CI: − 1.1–7.1, *p* = 0.152) or never-users (difference = − 3.9%, 95% CI: − 11.3–3.6, *p* = 0.306).

### Supplementary analyses

To explore whether prior smoking history influences respiratory outcomes among exclusive e-cigarette users, we conducted a subgroup analysis comparing individuals who had never smoked with those who previously smoked cigarettes (“switchers”). One participant was excluded due to missing smoking history data, resulting in a final sample of 519 exclusive e-cigarette users. Of these, 467 (90.0%) were former smokers, while only 52 (10.0%) had never smoked. A logistic regression model adjusted for age group, sex, deprivation level, presence of longstanding illness, doctor-diagnosed COPD, doctor-diagnosed asthma, self-rated general health, alcohol consumption status, and second-hand smoke exposure found no significant difference in the odds of wheezing between switchers vs. never-smokers (reference) (OR = 1.06, 95% CI: 0.43–2.61, *p* = 0.904). In contrast, exclusive cigarette smokers (*n* = 1732) were nearly evenly split between those who had previously used e-cigarettes (“switchers back to smoking,” *n* = 860, 49.7%) and those who had never vaped (*n* = 872, 50.3%). In this subgroup, a fully adjusted logistic regression model showed that switchers back to smoking had significantly higher odds of reporting wheezing compared to never-vapers (OR = 1.52, 95% CI: 1.19–1.94, *p* = 0.001).

**Fig. 3:**
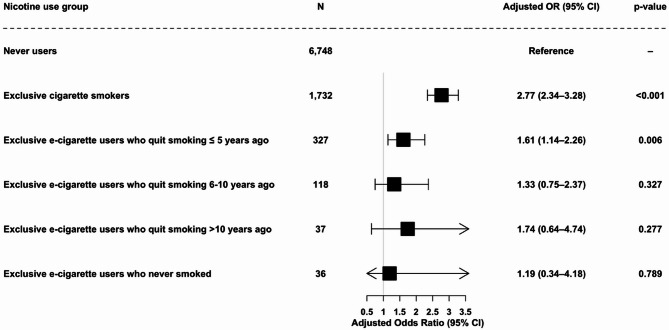
Multivariable logistic regression of wheezing by nicotine use categories: exclusive cigarette smokers, exclusive E-cigarette users by time since quitting smoking, and never users. Nicotine use was classified into six mutually exclusive groups: (i) current smokers (*n* = 1,732), (ii) exclusive e-cigarette users who quit smoking ≤ 5 years ago (*n* = 327), (iii) exclusive e-cigarette users who quit 6–10 years ago (*n* = 118), (iv) exclusive e-cigarette users who quit > 10 years ago (*n* = 37), (v) exclusive e-cigarette users who had never smoked (*n* = 36), and (vi) never-smokers who never vaped (reference group, *n* = 6,748). Models were adjusted for age group, sex, area-level deprivation, longstanding illness, doctor-diagnosed COPD, doctor-diagnosed asthma, self-rated general health, alcohol consumption, and second-hand smoke exposure. Two participants with missing data on time since quitting among exclusive e-cigarette users were excluded from categorisation

We next examined whether respiratory wheezing declined with increasing time since quitting smoking, using a six-category exposure specification: exclusive current smokers, exclusive e-cigarette users who quit smoking ≤ 5 years ago, 6–10 years ago, > 10 years ago, exclusive e-cigarette users who had never smoked, and never-smokers who never vaped (reference group). A multivariable logistic regression model was fitted, adjusting for age group, sex, area-level deprivation, longstanding illness, doctor-diagnosed COPD, doctor-diagnosed asthma, self-rated general health, alcohol consumption, and second-hand smoke exposure. Compared with never-smokers who never vaped, current smokers had almost threefold higher odds of reporting wheeze (OR = 2.77, 95% CI: 2.34–3.28, *p* < 0.001). Among exclusive e-cigarette users, those who had quit smoking within the past 5 years also had significantly elevated odds of wheezing (OR = 1.61, 95% CI: 1.14–2.26, *p* = 0.006). In contrast, e-cigarette users who had quit 6–10 years ago (OR = 1.33, 95% CI: 0.75–2.37, *p* = 0.327), those who had quit > 10 years ago (OR = 1.74, 95% CI: 0.64–4.74, *p* = 0.277), and those who had never smoked (OR = 1.19, 95% CI: 0.34–4.18, *p* = 0.789) did not differ significantly from never-smokers who never vaped (See Fig. 3).

### Sensitivity analyses

To further assess the robustness of the observed associations, a sensitivity analysis was conducted by excluding participants with doctor-diagnosed asthma or COPD (*n* = 1597), yielding a final analytical sample of 7403 adults. Within this subsample, 5680 were never users, 436 were exclusive e-cigarette users, and 1287 were exclusive cigarette smokers. The prevalence of wheezing in the past 12 months was 8.0% overall. In the unadjusted model, exclusive cigarette smokers had significantly higher odds of reporting wheezing compared to e-cigarette users (OR = 1.77, 95% CI: 1.29–2.42, *p* < 0.001), while never users had significantly lower odds (OR = 0.36, 95% CI: 0.27–0.50, *p* < 0.001). In the fully adjusted model, exclusive cigarette smokers had significantly higher odds of wheezing compared to exclusive e-cigarette users (adjusted OR = 1.60, 95% CI: 1.14–2.24, *p* = 0.006), whereas never users did not differ significantly (adjusted OR = 0.49, 95% CI: 0.20–1.18, *p* = 0.111). These findings are consistent with the main analysis.

As an additional sensitivity check, survey year was included as a covariate in all final models in this study to assess the potential influence of temporal variation. Including year did not result in any statistically significant changes in the estimated odds ratios. This suggests that the observed associations between nicotine product use and respiratory wheezing were consistent across survey years and not driven by time-related effects. Therefore, for clarity and parsimony, the results presented in this paper are based on models without year adjustment, as the inclusion did not meaningfully alter the findings or improve model fit.

A post hoc assessment indicated that the available sample size was sufficient to detect the observed differences in wheezing prevalence between nicotine use groups with very high statistical power. In particular, the study was well powered to detect differences between cigarette smokers and both e-cigarette users and never-users, while the comparison between e-cigarette users and never-users was also adequately powered (> 95%). These findings suggest that the sample size was adequate to detect clinically meaningful group differences.

## Discussion

This study demonstrates that exclusive cigarette smoking is associated with significantly higher odds of wheezing compared to exclusive e-cigarette use, with a clear dose–response gradient across smoking intensity. By contrast, the odds of wheezing among never-users did not differ significantly from those of exclusive e-cigarette users, although wide confidence intervals indicate some uncertainty. Taken together, these findings suggest that exclusive e-cigarette use does not materially elevate the odds of wheezing beyond that observed in never-users. Sensitivity analyses supported this interpretation, showing no significant differences in wheezing between never-users, exclusive e-cigarette users, or light smokers, though point estimates indicated a gradient with slightly higher risk among light smokers.

Our results are consistent with a growing body of research suggesting that exclusive e-cigarette use may impose a lesser respiratory burden than cigarette smoking. For instance, a recent analysis reported significantly lower odds of wheezing among exclusive e-cigarette users compared to cigarette smokers [25]. Similarly, another longitudinal research found no significant association between exclusive e-cigarette use and wheezing compared with never-users, while dual users and former smokers who vaped exhibited higher symptom prevalence [26]. Another recent study reinforced these trends, identifying exclusive cigarette use and polytobacco use as the strongest predictors of wheezing, with exclusive e-cigarette use linked to lower odds [27]. The VERITAS study of adults without prior tobacco use also noted that e-cigarette users reported slightly more frequent respiratory symptoms than non-users, but these differences were not clinically meaningful [28]. A systematic review of ten prospective studies in never-smokers further supports our results, finding no consistent link between exclusive e-cigarette use and severe respiratory outcomes, though mild associations with coughing or wheezing were occasionally noted [29].

While several studies suggest that exclusive e-cigarette use may pose less harm to respiratory health than smoking, other evidence presents a more uncertain picture regarding its effects relative to never-use. A systematic review assessed the respiratory effects of substituting cigarettes with e-cigarettes in human clinical studies, including sixteen studies and twenty publications identified [30]. The majority of included studies used spirometry to measure lung function, contributing 66 test outcomes in total. Among these, 65% showed no significant differences, while the remaining reported mixed effects: nine showing improvements and fourteen showing declines, none of which were clinically meaningful. However, the review highlighted substantial methodological concerns: ten studies were rated at high risk of bias, thirteen had evidence of reporting bias, and overall study heterogeneity precluded meta-analysis. Confidence in the conclusions was therefore rated as low. Despite the lack of strong evidence for additional harm, these findings underline the need for more robust, long-term clinical studies with adequate power to determine the true respiratory effects of e-cigarette substitution, particularly in comparison to never-users [30].

Furthermore, the observed differences in wheezing odds may reflect distinct pathophysiological mechanisms underlying cigarette smoking and e-cigarette use. Cigarette smoke delivers a complex mixture of over 7000 chemicals, including tar, carbon monoxide, and numerous carcinogens, which damage airway epithelium, trigger chronic inflammation, and promote goblet cell hyperplasia—factors that contribute to mucus hypersecretion and airway obstruction, key drivers of wheezing [31, 32]. In contrast, e-cigarette aerosol primarily contains nicotine, propylene glycol, vegetable glycerine, and flavourings, with fewer and less toxic constituents [15]. The lower probability of wheezing among e-cigarette users compared to cigarette smokers in our study suggests that these effects may be less severe or persistent, likely due to the absence of combustion-related toxins. Our adjusted analysis reinforces this interpretation, showing that moderate and heavy smokers had a significantly higher predicted probability of wheezing than e-cigarette users, even after controlling for socio-economic factors, chronic conditions and self-rated health. This supports the view that the respiratory harm from combustible tobacco is more pronounced. In addition, no significant differences were observed between e-cigarette users and never users or light smokers, indicating that e-cigarette use may pose relatively lower respiratory risk compared to heavier smoking. These findings highlight the need to distinguish between nicotine delivery methods in respiratory research and support the argument that switching to e-cigarettes may offer tangible respiratory benefits for individuals unable or unwilling to quit nicotine altogether [33].

Notably, some evidence suggests that e-cigarette use can cause transient respiratory irritation, particularly during the early stages of initiation [32]. Symptoms such as wheezing, cough, and shortness of breath have been reported, most often attributed to irritation and inflammation from inhaled propylene glycol and vegetable glycerine, especially among predisposed individuals such as those with allergic rhinitis or hay fever, a condition with high global prevalence [34, 35]. These early symptoms generally subside as users develop tolerance to the aerosol constituents, and they appear to have limited prognostic significance for long-term respiratory health [32]. In addition, it is plausible that some respondents in our study reported occasional wheezing not because of sustained e-cigarette use, but as a consequence of temporary airway perturbations following combustible cigarette cessation.

In the first few weeks to several months after quitting, many individuals experience a “healing cough” and wheeze, as regenerating cilia mobilise retained secretions and cough reflex sensitivity rebounds [34]. Although this rebound typically resolves within months, longer-term airway recovery can continue for several years for heavy smokers [35]. Consequently, individuals who quit within the past few years may remain at elevated risk of wheezing compared with never-users, regardless of current vaping status, due to incomplete structural and functional repair of the airways [36]. This mechanism may help explain why exclusive e-cigarette users as a group exhibited only a small, non-significant difference in wheezing compared with never-users, and why in our supplementary analysis, those who quit smoking within the past 1–5 years and now vape exclusively were more likely to wheeze than never-smokers or never-vapers. Importantly, this association was no longer evident among exclusive e-cigarette users who had quit more than 6–10 years ago, consistent with progressive airway recovery over time.

The dose–response relationship between cigarette smoking intensity and wheezing further supports a mechanistic gradient, with heavy smokers exhibiting the highest probability of wheezing. This likely reflects cumulative exposure to respiratory irritants and progressive airway remodelling, consistent with well-established evidence linking smoking quantity to diminished lung function, such as findings from the Framingham Heart Study [37]. By contrast, exclusive e-cigarette users, the reference group in our models, had a substantially lower adjusted probability of wheezing than smokers, though slightly higher than never-users. Although our study did not consider vaping intensity (e.g., puffs per day or nicotine strength) due to data limitations, recent evidence suggests that such behaviours may not significantly affect respiratory symptom burden [38]. Specifically, that study found that neither e-cigarette topography nor frequency of use predicted respiratory symptoms among exclusive vapers; rather, cigarette smoking status was the strongest predictor of symptom severity [38].

Our supplementary analysis further suggests that the elevated odds of wheezing among exclusive e-cigarette users may be concentrated in those who quit combustible smoking within the past 5 years. By contrast, e-cigarette users with longer cessation histories showed no significant difference from never-users, supporting the interpretation that airway recovery after smoking may take several years, even when nicotine use continues through vaping. Taken together, these findings indicate that combustible smoking remains the dominant factor shaping respiratory outcomes, with never-use representing the optimal respiratory health state. This underlines that while e-cigarettes may reduce harm relative to cigarettes, their respiratory impact is still greater than never-use and depends on exclusive use [39, 40].

The findings of our study carry important implications for public health policy and harm reduction strategies. Given the significantly lower odds of wheezing among exclusive e-cigarette users compared to cigarette smokers, alongside the dose-dependent relationship observed for smoking intensity and the minimal odds of wheezing among never-users, these results align with prior evidence suggesting that e-cigarettes may be a less harmful alternative for current smokers who are unable or unwilling to quit nicotine entirely [41–43]. Public health messaging could use this evidence to encourage complete switching from combustible tobacco to e-cigarettes among persistent smokers, while clearly emphasizing that never-use remains the safest option for respiratory health. Regulatory efforts should balance promoting harm reduction for adult smokers with stringent measures to curb youth access and marketing. Additionally, healthcare providers should be informed of these findings to guide evidence-based smoking cessation counselling, where e-cigarettes may serve as a transitional tool for some patients, though abstinence should be the ultimate goal.

This study has several methodological strengths. First, its use of a large, population-based sample enhances generalizability within the Scottish context, capturing a diverse range of demographic and health profiles. The focus on exclusive users of cigarettes or e-cigarettes, alongside never-users, is a key strength, reducing confounding from dual use or other tobacco products, which has clouded prior research. The comprehensive adjustment for confounders bolsters the robustness of the associations observed. The inclusion of smoking intensity categories and a test for trend provides a nuanced analysis, reinforcing the plausibility of a causal link between exposure level and outcome among smokers. Additionally, the binary definition of wheezing, based on recent (past 12 months) symptoms, offers a clinically relevant endpoint that is less prone to recall bias than lifetime prevalence, enhancing measurement reliability.

Despite these strengths, several limitations should be acknowledged. First, the cross-sectional design precludes causal inference, as temporality between nicotine use and wheezing cannot be established. Second, although the number of exclusive e-cigarette users was smaller than the cigarette-smoking group, a post-hoc power analysis indicated that the study remained adequately powered to detect the observed difference in wheezing, supporting the robustness of the main finding. Third, wheezing symptoms often resolve within a short period after cessation, which may bias the observed association. Fourth, reliance on self-reported data introduces potential biases: participants may underreport smoking or vaping due to social desirability, or misclassify wheezing without clinical validation. In addition, unmeasured confounders such as duration of e-cigarette use, device type, or environmental exposures (e.g. air pollution) could influence the results. Another limitation is that the Scottish Health Survey does not collect information on current use of pipes or cigars, preventing the exclusion of these users. While pipe and cigar use is relatively uncommon in the UK, not accounting for them may slightly limit the accuracy of our categorisation of nicotine product use. Finally, the absence of data on vaping intensity limits comparability with the smoking intensity analysis, leaving unanswered whether heavy vaping resembles the risks of light smoking or approximates the minimal risk of never-use.

Future investigations should focus on longitudinal study designs to better assess causal relationships, incorporating objective indicators such as spirometry and biomarkers of airway inflammation. Collecting detailed information on vaping behaviours, including usage frequency, nicotine concentration, device type, duration of use, and flavourings, would support a more nuanced evaluation of risk, especially relative to individuals who have never used nicotine products. Expanding the sample size of e-cigarette users with no prior smoking history could help clarify baseline risks, while conducting research in more diverse populations would enhance the generalisability of these findings beyond the Scottish context.

## Conclusion

In this population-based analysis of Scottish adults, exclusive e-cigarette use was associated with significantly lower odds of respiratory wheezing compared to exclusive cigarette smoking, even after adjusting for a broad range of demographic, health, and behavioural factors. The clear dose-response relationship observed across smoking intensity reinforces the strong respiratory burden associated with combustible tobacco use. Notably, the odds of wheezing among exclusive e-cigarette users did not differ significantly from those who had never used nicotine products, suggesting that exclusive e-cigarette use is associated with a lower likelihood of wheezing than smoking, and potentially a similar likelihood to never-use. However, longitudinal studies are needed to assess whether these differences reflect causal relationships.

## Data Availability

To download the dataset used in the analyses, please visit the UK Data Service: [https://ukdataservice.ac.uk/find-data/browse/health/](https:/ukdataservice.ac.uk/find-data/browse/health).
